# Salivary Macrophage Inflammatory Protein-1α Levels in Periodontitis Subjects Receiving Non-surgical Periodontal Therapy With and Without Photo-Biomodulation: A Prospective Interventional Controlled Trial

**DOI:** 10.7759/cureus.68980

**Published:** 2024-09-09

**Authors:** Fazal S Mujawar, Sameer A Zope, Girish Suragimath, Siddhartha Varma, Apurva V Kale

**Affiliations:** 1 Periodontology, School of Dental Sciences, Krishna Vishwa Vidyapeeth (Deemed to be University), Karad, IND

**Keywords:** biofilm, chemokine, diode laser, inflammation, periodontal healing, periodontal therapy, periodontitis, photobiomodulation, saliva, scaling and root planing

## Abstract

Introduction

Periodontitis is a complex interplay of bacterial infection and host response. Non-surgical periodontal therapy (NSPT) is effective but limited in thoroughly debriding challenging areas, often leading to recurrent bacterial colonization. Photobiomodulation (PBM), involving precise doses of laser photonic energy, has shown potential in modulating inflammation and promoting healing. This study investigates the impact of NSPT alone and NSPT combined with PBM on clinical parameters and salivary macrophage inflammatory protein-1α (MIP-1α) levels in periodontitis patients.

Methods

Ethical approval was obtained, and informed consent was secured from all participants. Sixty periodontitis patients aged 30-60 were selected and divided into two groups: Group 1 (NSPT alone) and Group 2 (NSPT with PBM). Clinical parameters including the plaque index (PI), gingival index (GI), probing pocket depth (PPD), and clinical attachment level (CAL) were assessed at baseline and 21 days post-treatment. Salivary MIP-1α levels were measured using an enzyme-linked immunosorbent assay (ELISA).

Results

Both groups showed significant improvements in the PI, GI, PPD, and CAL from baseline to 21 days post-treatment. Group 2 exhibited greater reductions in the GI, PPD, and CAL compared to Group 1. Salivary MIP-1α levels post-intervention showed reduction in both groups but were not statistically significant between the groups.

Conclusion

NSPT combined with PBM demonstrates greater clinical improvements than NSPT alone, highlighting PBM as an effective adjunctive therapy. Both groups showed a reduction in MIP-1α levels post-intervention, suggesting the potential of MIP-1α as a biomarker for periodontal disease activity.

## Introduction

Periodontal disease is an inflammatory condition affecting the supportive tissues of teeth. The etiology of periodontitis is intricate, predominantly characterized by the dynamic interplay between the subgingival bacterial biofilm and the host's immune response. Manifestations of periodontitis are contingent upon the intricate interplay of environmental and genetic influences [1]. The established approach for addressing periodontitis is non-surgical periodontal therapy (NSPT). NSPT holds the capacity to diminish the bacterial load and mitigate the microbe-induced inflammatory response, with the primary goal of eliminating the subgingival biofilm [2].

Scaling and root planing (SRP) plays a key role in the resolution of inflammation and the healing of periodontal pockets, primarily through the formation of a long junctional epithelium. The clinical efficacy of this technique is extensively documented, and presently, SRP is considered the 'gold standard' in the treatment of periodontitis [3]. Nevertheless, recurrent colonization by periodontal bacteria is common post-SRP. The ability to reach challenging areas like furcations, concavities, grooves, and distal regions of molars is often restricted in traditional SRP. Therefore, alternative approaches beyond conventional therapy may be necessary to thoroughly address these issues and promote successful periodontal healing. Supplementary therapeutic strategies that concentrate on incorporating additional therapies to augment the outcomes of traditional treatment methods, such as laser therapy, have been suggested [4]. Recently, there has been a burgeoning interest in employing diode lasers for periodontal treatment. Research has demonstrated the safe applicability of diode lasers in periodontal practice.

Photobiomodulation (PBM), formerly recognized as low-level laser therapy, represents a therapeutic treatment approach centered on the utilization of precise doses of laser photonic energy [5]. The mechanism of action of PBM involves the modulation of the inflammatory process, changes in nerve conduction and excitation in peripheral nerves, and stimulation of endogenous endorphin production, resulting in pain reduction. Additionally, PBM inhibits the release of arachidonic acid, which typically acts on damaged cells to generate metabolites interacting with pain receptors. It is proposed that PBM influences cellular behavior by impacting the mitochondrial respiratory chain or membrane calcium channels, thereby facilitating collagen synthesis, angiogenesis, and the release of growth factors all of which contribute to an accelerated process of wound healing [6]. In periodontal disease, the balance between pro- and anti-inflammatory responses is directed toward proinflammatory activity [7]. Macrophage inflammatory protein 1α (MIP-1α) serves as a key chemokine involved in inflammatory response. This chemokine contributes to the recruitment and activation of immune cells, particularly macrophages, exacerbating the inflammatory cascade in periodontitis. Tissue destruction and bone resorption in the periodontal microenvironment are associated with the upregulation of MIP-1α [8].

Currently, there is limited data on salivary MIP-1α levels in periodontitis patients undergoing NSPT combined with PBM. No studies have specifically evaluated this, or any available studies may not have been published or identified in the literature search. We hypothesized that NSPT combined with PBM would result in greater improvements in clinical parameters and a more substantial reduction in salivary MIP-1α levels compared to NSPT alone. Therefore, this study aims to analyze salivary MIP-1α levels pre- and post-NSPT, and NSPT with PBM, in periodontitis patients. This investigation is essential for understanding the potential of MIP-1α as a salivary biomarker for periodontal disease activity.

## Materials and methods

Study design

This single-blinded, prospective interventional, parallel-group clinical trial was conducted in the Department of Periodontology at Krishna Vishwa Vidyapeeth, Karad. Before beginning the study, the stages were thoroughly explained to the patients, and written informed consent was obtained. Patients were recruited from June 30, 2024 to July 15, 2024.

Ethical consideration

The ethical clearance was obtained from the institutional ethical board (Protocol No. 012/2022-2023). The Consolidated Standards of Reporting Trials (CONSORT guidelines) were used to design, analyze, and interpret this study. Also, the study is registered under clinical trial registry- India with registration no. CTRI/2024/06/069189.

Selection criteria

Selection criteria for both groups include systemically healthy patients aged 30 to 60 years with generalized Stage 2 and Grade B periodontitis as per AAP criteria 2017; patients were selected to provide a clear representation of moderate periodontitis with a balanced progression rate, making them ideal for studying disease characteristics and minimizing variability in severity and disease progression. Subjects with systemic diseases, recent infections, pregnant or lactating women, those who have undergone periodontal therapy within the last six months or used medications known to influence periodontal tissues, and participants using tobacco were excluded from the study.

Sample size estimation

A power analysis was established by G*Power version 3.0.1 (Franz Faul Universitat, Kiel, Germany). The total calculated sample size of 40 subjects (20 subjects - Group 1, 20 subjects - Group 2) would yield 80% power to detect significant differences, with an effect size of 0.92 and a significance level of 0.05. Considering the dropouts from the study, the sample size was taken as 60 subjects (30 subjects - Group 1, 30 subjects - Group 2)

Clinical examination

The clinical evaluations were performed at two time points, initially at baseline prior to intervention, and subsequently 21 days following treatment. To maintain consistency and reliability of the data, a single trained examiner carried out all measurements and recordings. The clinical periodontal parameters including, plaque index (PI), gingival index (GI), probing pocket depth (PPD), and clinical attachment level (CAL) were evaluated for the enrolled subjects. Midbuccal, distobuccal, mesiobuccal, and palatal sites in each tooth were recorded for PI. The buccal, mesial, distal, and lingual gingival areas were examined for the GI. The PPD and CAL were measured in millimeters and were assessed in all the teeth at six sites and the CAL was calculated from the cemento-enamel junction to periodontal pocket base. An orthopantomogram (OPG) was taken for each patient and the bone loss was assessed.

Intervention

Subjects were randomly allocated into treatment groups using the lottery method. In Group 1, all patients received SRP performed by a single operator under local anesthesia. This was done manually with area-specific Gracey curettes and supplemented by an ultrasonic scaler (EMS PM100) in a single appointment. Additionally, standard oral hygiene instructions were provided to the patients. In Group 2, laser PBM was performed after SRP using a 980 nm diode laser (Photon plus; Zolar Technology Co Inc., Canada) with 100 mW output power in continuous wave mode. The laser was applied to four points: the interdental papilla on the labial/buccal, lingual/palatal aspect, mesial, and distal aspect of the tooth. The probe was positioned perpendicular to the long axis of the tooth in a noncontact mode at a 3 mm distance from the mucosa for 10 seconds per point, amounting to a total dose of 8 Jules.

Sample collection

Two milliliters of unstimulated whole saliva were collected by following the circadian rhythm, between 10 am and 12 pm, two hours after the last meal. To prevent contamination, participants were asked to rinse their mouths thoroughly with distilled water before collection. They were instructed to refrain from talking and asked to accumulate saliva in their mouth before drooling it into a collection vessel until the desired volume was collected. The saliva samples were then centrifuged at 5000 rpm at 2-8°C for five minutes (Remi R-8C centrifuge, India) to remove any cell debris. Approximately 0.5 ml of the supernatant was transferred into labeled 1.5 ml Eppendorf tubes and stored at the Immunology Laboratory of Krishna Vishwa Vidyapeeth while maintaining the cold chain (-70 °C temperature) until the tests were performed. The saliva collection was done at baseline before intervention and post-21 days.

Biochemical examination

Levels of salivary MIP-1α were determined using an MIP-1α enzyme-linked immunosorbent assay (ELISA) kit (Abbkine CCL3 ELISA kit). The assay was carried out using an ELISA reader (LisaQuant, Department of Microbiology, KVV, Karad, India) according to the instructions and directions of the manufacturer. Before its use, the components of the kit were allowed to equilibrate at room temperature.

Statistical analysis

**Figure 1:**
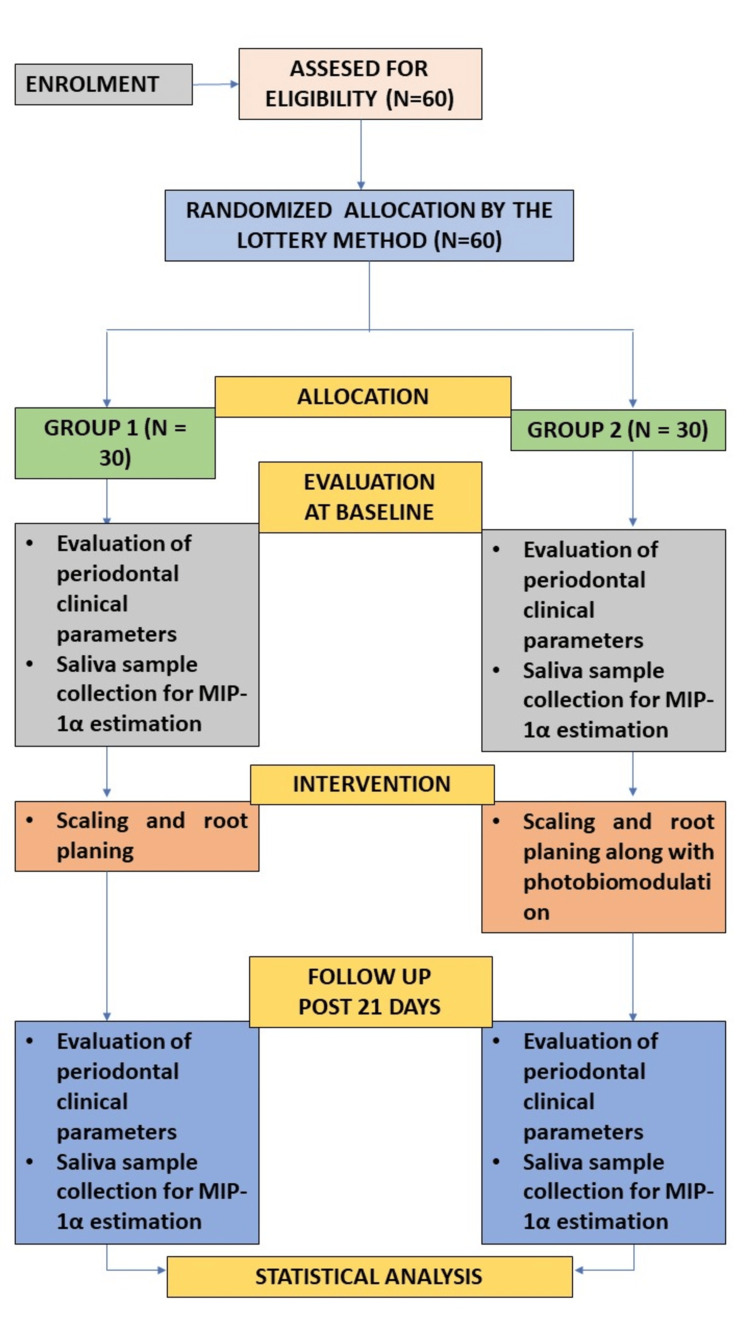
Summary of the study protocol

All statistical analyses were conducted using IBM SPSS Statistics for Windows, Version 25 (Released 2017; IBM Corp., Armonk, New York, United States). For intragroup comparisons of the PI, GI, CAL, PPD, and salivary MIP-1α levels between baseline and 21 days, a paired t-test was used. Intergroup comparisons of clinical and biochemical parameters between the groups at baseline and 21 days were performed using an independent samples t-test. The significance level was set at p < 0.05. The steps of the methodology are explained in Figure 1.

## Results

Table 1 illustrates the mean PI values at baseline and on the 21st day. For Group 1, the mean PI was 1.3 at baseline and decreased to 0.7 on the 21st day. In Group 2, the mean PI was 1.5 at baseline and reduced to 1.1 by the 21st day. Both groups exhibited a significant reduction in PI scores when compared to their baseline values.

**Table 1:** Intragroup comparison between the two groups in terms of the plaque index *p<0.05: statistically significant, statistical test used: paired t-test. NSPT: Non-surgical periodontal therapy; PBM: photobiomodulation

Plaque index	Mean and standard deviation	p-value
NSPT (Baseline)	1.3 ± 0.43	p= 0.039*
NSPT+ PBM (Baseline)	1.5 ± 0.53	
NSPT (21 days)	0.7 ± 0.4	
NSPT+ PBM (21 days)	1.1 ± 0.29	

Table 2 depicts the average GI values at baseline and on the 21st day. In Group 1, the mean GI was 1.2 at baseline and 0.7 on the 21st day. In Group 2, the mean GI was 1.5 at baseline and 1.0 on the 21st day. Statistical analysis revealed a significant decrease in mean GI values from baseline to the 21st day in both groups (p<0.001).

**Table 2:** Intragroup comparison between the two groups in terms of the gingival index *p<0.05: statistically significant, statistical test used: paired t-test. NSPT: Non-surgical periodontal therapy; PBM: photobiomodulation

Gingival index	Mean and standard deviation	p-value
NSPT (Baseline)	1.2 ± 0.44	p = 0.004*
NSPT+ PBM (Baseline)	1.5 ± 0.5	
NSPT (21 days)	0.7 ± 0.38	
NSPT+ PBM (21 days)	0.9 ± 0.26	

The average PPD values at baseline and on the 21st day are shown in Table 3. In Group 1, the mean PPD was 5.6 at baseline and 3.8 post-intervention. In Group 2, the mean PPD at baseline was 5.4 and 2.8 on the 21st day. Significant reductions in the PPD were observed from baseline to the 21st day in both groups (p<0.001).

**Table 3:** Intragroup comparison between two group in terms of probing pocket depth *p<0.05: statistically significant. Statistical test used: paired t-test. NSPT: Non-surgical periodontal therapy; PBM: photobiomodulation

Periodontal pocket depth	Mean and standard deviation (mm)	p-value
			NSPT (Baseline)	5.6 ± 0.77	p= 0.002*
			NSPT+ PBM (Baseline)	5.4 ± 0.93	
NSPT (21 days)	3.8 ± 0.85				
NSPT+ PBM (21 days)	2.8 ± 0.81				

Table 4 displays the average values of the CAL at baseline and on the 21st day. In Group 1, the mean CAL was 3.6 at baseline and decreased to 1.9 on the 21st day, while in Group 2, the mean CAL at baseline was 3.6 and reduced to 1.1 on the 21st day. Intragroup analysis revealed a statistically significant difference from baseline to the 21st day in both groups (p<0.001).

**Table 4:** Intragroup comparison between the two groups in terms of the clinical attachment level *p<0.05: statistically significant. Statistical test used: paired t-test. NSPT: Non-surgical periodontal therapy; PBM: photobiomodulation

Clinical attachment level	Mean and standard deviation (mm)	p-value
			NSPT (Baseline)	3.6 ± 0.72	p= 0.034*
			NSPT+ PBM (Baseline)	3.6 ± 0.68	
NSPT (21 days)	2.0 ± 1.19				
NSPT+ PBM (21 days)	1.1 ± 0.77				

Table 5 presents the mean values of salivary MIP-1α. In Group 1, the mean salivary MIP-1α level was 223.29 pg/ml at baseline and decreased to 208.92 pg/ml on the 21st day. In Group 2, the mean salivary MIP-1α level was 227.11 pg/ml at baseline and 210.42 pg/ml on the 21st day.

**Table 5:** Intragroup comparison between the two groups in terms of MIP-1 alpha levels at baseline and post-21 days *p<0.05: statistically significant. Statistical test used: paired t-test NSPT: Non-surgical periodontal therapy; PBM: photobiomodulation

MIP-1 alpha levels	Mean and standard deviation (pg/ml)	p-value
			Group 1 (NSPT- Pre)	223.29 ± 32.16	p= 0.041*
Group 1 (NSPT -Post)	208.92 ± 17.71				
Group 2 (NSPT+PBM Pre)	227.11 ± 30.78	p= 0.010*			
Group 2 (NSPT +PBM Post)	210.43 ±13.24				

Table 6 presents an intergroup comparative analysis, revealing a significantly greater reduction in PI scores in Group 1 compared to Group 2 (p<0.05). The table also shows that the combination of NSPT and PBM resulted in a greater reduction in GI scores compared to NSPT alone. Moreover, the post-intervention intergroup comparison indicated a significant reduction in the PPD in the group receiving both NSPT and PBM compared to the group receiving NSPT alone (p<0.001). Additionally, it was observed that the CAL gain was more pronounced in Group 2 than in Group 1 (p<0.001). However, the intergroup comparison of MIP-1α levels showed a reduction, but the difference between the groups was not statistically significant (p>0.05).

**Table 6:** Intergroup comparative statistics in both groups in terms of various parameters p>0.05 – Statistically not significant difference, *p<0.05 – statistically significant, Statistical test used: independent sample t-test

Parameters	Mean difference Between NSPT post 21 days (Group 1) Vs. NSPT +PBM 21 days (Group 2)	p-value
			Plaque index	0.357	p =0.001*
						Gingival index	0.197	p = 0.063

## Discussion

Periodontitis is an inflammatory disorder characterized by the interaction between pathogenic bacteria and the immune and inflammatory responses of the host. A complex network of cytokines is involved in the inflammatory and immune responses in the periodontal tissues during the progression of periodontal disease [9].

Macrophage inflammatory protein-1 α (MIP-1α/CCL3) is a chemotactic chemokine secreted by macrophages. MIP-1α/CCL3 serves multiple biological roles, including the recruitment of inflammatory cells, promotion of wound healing, inhibition of stem cells, and maintenance of effector immune response. Moreover, it stimulates bone resorption cells and directly contributes to bone destruction. Elevated secretion of MIP-1α/CCL3 occurs at sites of inflammation and bone resorption [10]. Increased production of MIP-1α. Also, it is found to be one of the chemokines copiously expressed in inflamed gingival tissue and by oral epithelial keratinocytes [11]. Salivary levels of MIP-1α/CCL3 strongly correlate with clinical parameters of periodontal disease and reflect disease severity, as their levels start to reduce with a decrease in disease severity [8]. Additionally, these levels are reflective of the response to therapy. The interrelationship between chronic periodontitis and MIP-1α levels in GCF, serum, and gingival tissues has been investigated in a considerable number of studies [12]. However, to the best of our knowledge, this is the first clinical interventional study that has assessed the impact of non-surgical periodontal intervention on MIP-1α levels in saliva.

PBM is recommended for its pain-reducing, wound-healing promoter, and anti-inflammatory effects [13]. It is suggested that PBM induces changes in cellular behavior through its effects on the mitochondrial respiratory chain or membrane calcium channels. This process can promote collagen synthesis, angiogenesis, and the release of growth factors. Ultimately, these effects contribute to accelerating the healing of wounds [14], for PBM, the diode laser can be used safely in periodontal practice [15]. Hence in this study, PBM was used as an adjunct to NSPT to evaluate and compare salivary MIP-1α levels in periodontitis. The beneficial effects of SRP combined with the incorporation of plaque control measures in the treatment of periodontitis have been well documented [16]. This includes reduction of clinical inflammation, microbial shifts to a less pathogenic subgingival flora, reduction of PPD, and gain of clinical attachment.

Nisha et al., in their biochemical analysis of MIP-1α assessed in saliva, revealed that the lowest mean value of MIP-1α was found in the healthy periodontium group and it significantly increased with progression in stages of periodontitis in the periodontitis group [17]. Al-Sabbagh et al. found that saliva from periodontitis patients contained higher concentrations of MIP-1α than healthy controls [18]. The present study reported a marked reduction in periodontal parameters including PI, GI, PPD, and CAL after NSPT alone. The results were consistent with the study done by Sanz et al., which showed that NSPT significantly improved CAL levels and reduced PPD in periodontitis [19]. Tawfig et al. evaluated changes in the GI after SRP treatment from baseline and found a significant improvement in the SRP group at three months [20]. These results are in accordance with the present study as this study reported a significant reduction in the PPD, PI, and GI and also significant CAL gain after NSPT alone.

The present study found a significant reduction in salivary MIP-1α levels in both groups at baseline and after 21 days. However, there was no significant reduction observed between the two groups postoperatively. Since there are no studies available on the comparison of salivary MIP-1α levels in NSPT and NSPT with the PBM group in generalized stage II and grade B periodontitis, a direct comparison of our results is not possible. Nagireddy et al. found that GCF and serum MIP-1α levels increased proportionally with the progression of periodontal disease and decreased after NSPT treatment [21].

In the present study, the PI score showed a statistically significant reduction from baseline to after 21 days in both groups. Also, the PI score was reduced more in the NSPT group than in the NSPT with PBM group. This finding is in accordance with the study by De Micheli et al. [22].

The current study reported statistically significant decreased GI levels in the PBM group compared with SRP alone suggesting that the adjunctive use of PBM is more beneficial than SRP alone in reducing gingival inflammation. These results are in accordance with the study by Qadri et al. and Gandhi et al. [23,24]. This improvement could be a result of an increase in the anti-inflammatory cytokine levels and an increase in microcirculation by low-level laser irradiation.

The intragroup comparison in the present study revealed a significant reduction in the mean PPD and CAL gain in NSPT alone and the NSPT with PBM group pre and post-intervention. The intergroup comparative analysis demonstrated a reduction in the PPD and CAL gain was more in the NSPT with PBM group than in NSPT alone after 21 days but the difference was not significant. Similar results were reported by Angiero et al. in their study where both the SRP group and SRP with PBM group had shown clinical improvement over the control group, but with no statistically significant difference between each other [25]. Several other studies conducted by Petrovic et al. and Gandhi et al. reported a significantly higher reduction in clinical and microbiological parameters in periodontitis subjects two months postoperatively after NSPT along with PBM [24,26]. One probable reason could be that laser irradiation reduces PGE2 and stimulates cellular ATP [27].

In our study, we observed a significant reduction in clinical parameters, including GI, PD, and CAL, following NSPT with PBM compared to NSPT alone. These findings differ from those reported by Özdemir et al., who found no difference in the GI between the PBMT + SRP group and the SRP alone group [28]. Similarly, Euzebio Alves et al. reported no significant reduction in clinical parameters after NSPT with PBM compared to NSPT alone [29]. Also, Chandra et al. observed no significant changes in the GI, PD, or CAL gain between the laser PBM group and the NSPT group [30]. The discrepancies between our results and those of previous studies could be due to differences in study design, patient selection, laser parameters, or follow-up duration, which may have influenced the outcomes in our sample.

The study presents several limitations that affect the generalizability of its findings. Firstly, the relatively small sample size limits the ability to extrapolate the results to a broader population. Future research with larger cohorts is necessary to validate our findings and provide a more comprehensive understanding of the effects of PBM combined with NSPT. Additionally, the study evaluated the impact of a single application of PBM, which may not fully capture the treatment's efficacy over time. The potential benefits of repeated PBM sessions warrant further exploration. Furthermore, the study's design included only one follow-up appointment, restricting the assessment of the long-term effects of the intervention. Long-term studies are needed to evaluate the durability of the treatment effects and whether the observed benefits are sustained over extended periods. Lastly, the lack of blinding for the examiner introduces the possibility of observer bias, which could affect the objectivity of the assessments and the interpretation of results. Future research should incorporate blinding procedures to minimize such biases and enhance the reliability of the findings.

## Conclusions

Both NSPT combined with PBM and NSPT alone have demonstrated improvements in clinical and biochemical parameters following intervention. The NSPT-only group showed a significantly better plaque index, while the NSPT with PBM group experienced more notable improvements in the PPD and CAL. No significant differences were observed between the two groups in terms of GI and salivary MIP-1α levels. Conventional NSPT is effective for managing periodontitis, and PBM proves to be a valuable adjunctive therapy. However, the addition of PBM does not appear to provide a significant advantage over NSPT alone regarding MIP-1α levels. MIP-1α remains a promising diagnostic and prognostic marker for periodontal disease progression and could serve as a useful salivary biomarker for evaluating treatment outcomes. Future research should focus on larger and more comprehensive studies to enhance the reliability and generalizability of these findings.
